# Navigational Strategies and Their Neural Correlates

**DOI:** 10.1007/s41745-017-0053-1

**Published:** 2017-11-24

**Authors:** Deepa Jain, Indraja R. Jakhalekar, Sachin S. Deshmukh

**Affiliations:** Centre for Neuroscience, Indian Institute of Science, Bangalore, India

**Keywords:** Hippocampus, Medial entorhinal cortex (MEC), Lateral entorhinal cortex (LEC), Spatial navigation, Path integration, Landmarks

## Abstract

Animals depend on navigation to find food, water, mate(s), shelter, etc. Different species use diverse strategies that utilise forms of motion- and location-related information derived from the environment to navigate to their goals and back. We start by describing behavioural studies undertaken to unearth different strategies used in navigation. Then we move on to outline what we know about the brain area most associated with spatial navigation, namely the hippocampal formation. While doing so, we first briefly explain the anatomical connections in the area and then proceed to describe the neural correlates that are considered to play a role in navigation. We conclude by looking at how the strategies might interact and complement each other in certain contexts.

## Introduction

Navigation is a seemingly arduous task for many animals, as it involves travelling over vast expanses of land or water. Examples of such large-scale movements include the migration of monarch butterflies up to 4000 km within North America^80^ and that of the Arctic tern from boreal Arctic grounds to the Southern Ocean and back, a journey of over 70,000 km on average^27^. African straw-coloured fruit bats can fly up to 88 km while foraging^29^. What guides these animals as they traverse over such long distances? How can they follow such specific paths? Is there any common strategy adopted by such a diverse set of animals to be able to make long journeys, without losing track of their location?

Animals use two broad classes of inputs to navigate—allothetic and idiothetic. Allothetic cues are forms of sensory information derived from the environment (external to the body of the animal) and are picked up by many different sensory modalities, including the visual, the auditory and the olfactory systems. Idiothetic cues, by contrast, are self-referential forms of information generated when the animal undertakes movement. Examples of idiothetic cues include proprioception (the sense of position of one’s body parts) and efference copy (an internal copy of movement-generating signals produced by the motor system). Additionally, sensory flow, which is the apparent motion of sensory stimuli caused by relative motion between the observer and the surroundings, is also considered to be an idiothetic cue, since it is generated during movement. Olfactory, optic, auditory and somatosensory modalities contribute to this sensory flow. Using different combinations of these inputs, animals navigate following various kinds of strategies, which can broadly be divided into two classes.

The first of these is path integration. It involves the use of self-generated cues to keep track of the displacement from one’s starting position, to estimate the current position. By integrating its velocity (speed and direction of travel) as deduced from idiothetic cues, an animal can gauge its current location relative to its starting point, or some other stable reference point. Many animals use this strategy to create a ‘homing vector’, which they can utilize to travel back home, in an approximately straight-line path, after an outbound journey. On account of this characteristic, path integration is also called dead reckoning (an abbreviation of deduced reckoning)^36^. Path integration is commonly used in the case of short trajectories and can function as an independent strategy. In case of longer trajectories, though, the errors in integration at each step of the journey add up and lead to error-prone position estimates, reducing the utility of this strategy^94^. However, if supplemented by information from allothetic cues, this error can be reduced.

One example of dead reckoning is seen in the case of mother gerbils retrieving pups that have been displaced by researchers from their nests; the mothers, on finding their pups, pick them up, and follow a straight-line path back to their original starting point, even under the cover of complete darkness^58^. Another classic example of path integration is seen in the case of the desert ant (*Cataglyphis fortis*), which, after following a tortuous path from its nest in search of food, heads straight back home after finding food^64^. However, path integration is not exclusively used in homebound journeys; it is also used by desert ants to navigate to previously visited food sources^18^.

Not surprisingly, humans navigate using path integration as well. One example of this was demonstrated in the case of a study^52^ in which blindfolded adult humans were asked to perform either simple locomotion tasks, which involved estimating and physically reproducing distance measurements, or a more complex task, in which the participants were guided along two arms of a triangle, and then asked to return to the starting point. Participants were able to perform both sets of tasks with reasonable accuracy, although systematic errors did occur. In real-life situations, path integration is commonly used by sailors in combination with landmarks to navigate over large distances—even Christopher Columbus used dead reckoning in his voyages^36^.

The other class of navigational strategies commonly utilized by animals is based on the use of landmarks, which are defined as cues that have some fixed relationship with space (i.e., they are stable in the environment), and additionally, have informative and salient features. They act as points of reference and play an important role in navigation, since they serve as anchor points during the formation of a map of an environment, or could be used for identifying a particular route^13^.

Many strategies including beaconing, route following and piloting use stable objects in the environment; we cover each of these briefly here.

In beaconing, an animal uses sensory features of a distal object to navigate to it directly, or to a goal that is in the direction of the object. The distal object, thus, acts like a beacon, in that it provides a salient signal towards which the animal moves (hence giving this strategy its name)^13^. The features of the beacon used by the animal to navigate towards it could take the following forms:

1. Visual features, as in the case of small-eyed ants (*Leptothorax albipennis*) that can navigate between nest sites using a prominent visual beacon^54^.
2. Auditory cues, for example, when big brown bats use a swamp frog chorus as an auditory beacon^10^.
3. Olfactory features, seen, for example, in Pacific salmon, which migrate from their feeding grounds in the Pacific Ocean to the rivers in which they were born. During the migration, the fish swim towards a higher concentration of the odour of their homestream water^25^.

In beaconing, the path followed by the animal is often the shortest one possible, being a straight-line path. Since only a single beacon is involved, it is computationally simple, and consequently, is thought to be one of the most fundamental navigation strategies used—this is borne out by the fact that it is one of the first mechanisms to be seen during development in humans^50^. Mice learning to home in an experimental arena first learn to navigate by beaconing^2^.

A second strategy is route following, which involves using a series of landmarks or well-marked routes to navigate to a target. It is also a simple navigational strategy, as it merely involves identifying cues and following their location^93^. One example of route following is seen in the foraging journeys of the limpet *Siphonaria alternata*—the route is marked by mucous trails laid down by the limpets as they travel^20^.

Map based strategies can use a combination of path integration and landmarks to navigate. A topographical map represents aspects of the environment, such as the shapes, sizes and relative locations of different features in space. Animals are thought to have spatial maps in their brains (see below for neural correlates of these maps). Over the years, descriptions of the features and functions of these maps and their neural correlates have grown in complexity. Tolman^88^ noted that animals learn not just the path leading to food but the location of food relative to the starting point. He proposed that they create a comprehensive map of space in which relative locations of different constituents of the map are stored and can be used flexibly for performing tasks different from the ones the animal learned the map in. He called it a cognitive map and extended this concept to include all kinds of abstract maps (e.g. those encoding relationships between people, emotions, experiences, etc.). Using evidence from behaviour, lesion and physiological studies, O’Keefe and Nadel^69^ linked the cognitive map to the hippocampus. They proposed that the hippocampal spatial map provides a framework for organizing items and events of experience, creating a cognitive map. This conception borrows Tolman’s idea of spatial maps being a comprehensive representation of spatial relationships between different locations, but also modifies the idea of the cognitive map itself. In this new formulation, the spatial map is of primary importance, and experience and knowledge is supposed to be organized in this framework, unlike Tolman’s cognitive maps, which envisage direct relationships between all kinds of abstract ideas, without requiring space as an intermediary.

One navigational strategy that utilizes a cognitive map is piloting. When an animal uses a piloting strategy to navigate, it is thought to utilize such a cognitive map to identify the location of the target, and to move either towards it or steer away from it, depending on whether the target is a goal or an obstacle, respectively. In identifying the location of the target, the animal does not use any cues emanating from it (i.e., the target is undetectable to the animal). Instead, it infers the position of the target by using stimuli whose geometric relation with the target is known and then adopts an appropriate orientation—facing towards the target, if it is a goal, or away from it, if it is an obstacle—as it initiates movement^36^. An interesting example of piloting is seen in the case of hoverfly hovering^19^. Male hoverflies hover in the air in a particular volume of space termed as the station of the fly. By definition, this station does not have any distinctive features of its own, and it can be described only with respect to landmarks. When a hoverfly attempts to return to its station after an outward foraging journey, it uses piloting to infer the position of its station with respect to the surrounding landmarks^36^.

Many animal species use a combination of these varied navigational strategies—for instance, ants often use a combination of path integration and route following in their foraging journeys. Nevertheless, it appears that some animals primarily use path integration to determine their position, even while navigating through familiar environments. Only occasionally in its traversal does the animal verify its position by using its perception of the surrounding landmarks (i.e., allothetic cues) to determine its position, and comparing this estimate with that which it has obtained by path integration (in navigational terms, this is termed as ‘taking a fix’)^36^. In other words, from time to time, the animal uses position estimated from landmarks to update its path integration estimate, in the process, resetting the path integrator. For example, golden hamsters reset their path integrator systems after navigating in the darkness, by using visual landmarks in their vicinity^28^, and desert ants that have ventured from their nests on a foraging expedition reset their path integrators when they are forcibly transferred back inside their nest^46^.

However, the role that path integration plays in complementing landmark-based navigation has not been explored—theoretically, it is possible that path integration can help in calibrating the size of visual landmarks when viewed for the first time. The visual angle subtended by a landmark in combination with knowledge of the size of the landmark can be used to estimate distance from the landmark. Since the animals would have no expectation of the size of the novel landmarks, they would be unable to position themselves in space using these objects. Instead, they could possibly use information from the path integration system to derive an estimate of the landmarks’ size, whereupon they could use this information in navigation. In this manner, path integration could assist in calibration of landmark-based navigation systems.

These behavioural studies prompted an interest in identifying the part/parts of the brain that govern navigation, and that help in the creation of a cognitive map. Lesion studies, wherein one disables a certain part of brain to see what activities of the animal get affected in absence of/damage to the that part, showed that damage to the hippocampus and associated areas affected abilities of rats to learn spatial tasks(Morris et al.^60^; see O’Keefe and Nadel^69^ for an extensive review of early experiments implicating hippocampus in spatial navigation). Around the same time, O’Keefe and Dostrovsky^68^ recorded neuronal activity from the hippocampus, which led to the discovery of place cells (see below). Consequently, the hippocampus was considered to be associated with navigation and spatial memory.

We proceed now to describe the anatomy of the different brain regions involved in spatial navigation and their connectivity patterns. We then move on to a review of the main categories of spatially modulated cells and their properties, which are the neural correlates of navigation. Finally, we review the ways in which landmark-based navigation and path integration may interact with and complement each other—on the one hand, in terms of correcting for errors in navigation, and on the other, in assisting in initial calibration for navigation.

## Anatomical Connectivity Patterns

Since studies of spatial navigation often involve several interconnected brain regions, understanding the nature and strength of their connectivity patterns is essential to appreciate how the neural correlate of space transforms as it flows from one brain region to another. Hence, we first look at anatomy of the regions of interest.

**Figure 1:**
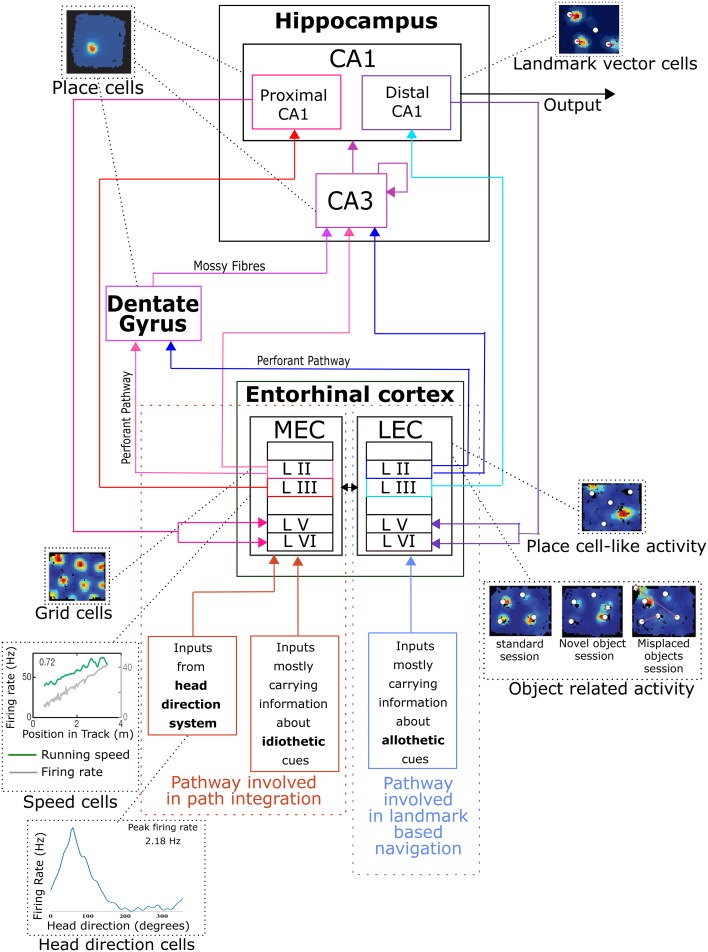
The central part of figure is a block diagram of areas of the hippocampal formation that we discuss in the review. Major connections between these areas are indicated by arrows. The figure also contains plots showing firing characteristics of different cell types. Every such plot is connected by a dotted line to the block representing the brain regions the corresponding cell type is found in. Rate maps are shown for place cells (reprinted from Deshmukh and Knierim^23^), grid cells (reprinted from Deshmukh and Knierim^22^), landmark vector cells (reprinted from Deshmukh and Knierim^24^), cells showing place-cell like activity and object related activity in the LEC (reprinted from Deshmukh and Knierim^22^) are shown. A rate map is created by superimposing the firing rate of a neuron at given instant of time with the location of animal in the environment at the same instant. The firing rate is colour-coded with warmer colours representing higher firing rate and cooler colours representing lower firing rate. For LEC rate maps, the white circles depict standard objects in their standard locations while a white star depicts a novel object in the novel object session and the misplaced object in misplaced object session, where it is connected to its standard location with magenta lines. For the HD system, the plot of firing rate as a function of HD is shown (Rajat Saxena, Warsha Barde and Deshmukh, Unpublished data). The selectivity of a HD cell is evident from the higher firing rate in a narrow range of head directions. For speed cells (reprinted from Kropff et al.^48^), the plot shows increase in firing rate of a speed cell which is correlated with increase in the speed of the animal.

The hippocampus^68^^,^ 69, the EC^22^^,^ 40, the subiculum^72^^,^ 86, the pre- and the para-subiculum^8^^,^ 85 and the dentate gyrus^43^ have been implicated in spatial navigation. We describe some of these areas briefly in this section, to give a picture of the intricate network involved (for more details, refer to Amaral and Lavenex^3^). For ease of understanding, we have organized this section in terms of the pathways of information flow (see Fig. 1 for anatomical connectivity and neural correlates to be discussed in subsequent sections).

### Entorhinal Cortex (EC)

The EC is the primary source of cortical information to the hippocampus^97^. Named so because it is partially enclosed by the rhinal sulcus (entorhinal = inside rhinal), the EC can be divided into two subdomains, which differ in their input–output connectivity patterns^98^. One sub-division, the medial entorhinal cortex (MEC), receives stronger inputs from the postrhinal cortex, the presubiculum, and the occipital and the retrosplenial cortices^97^^,^ 98. The other sub-division, called the lateral entorhinal cortex (LEC), receives stronger inputs from the perirhinal, the olfactory and the insular cortices and the amygdala^11^^,^ 98. The differential inputs to these two regions suggest that the MEC is involved in processing idiothetic cues (as its inputs are part of the dorsal “where” stream that processes location information derived from visual input) and that the LEC, in contrast, plays a role in processing allothetic cues (as its inputs are part of the ventral “what” pathway of visual information processing, which deals with object identity^91^).

Superficial layers II and III of both the LEC and the MEC send inputs to the dentate gyrus, the hippocampus and the subiculum, but there is a difference in the regions targeted by cells from the two layers. While layer II MEC and LEC cells target the dentate gyrus, and the CA3 sub-region of the hippocampus (for more information on the CA1-3 sub-regions, see Sect. 2.3), layer III cells send inputs to CA1 and subiculum in such a manner that MEC layer III cells project to proximal CA1 and distal subiculum, while LEC layer III cells send their inputs to distal CA1 and proximal subiculum. Outputs from the subiculum and CA1, segregated along the proximal–distal axis, feed back to the deep layers V and VI of the same regions of the EC from which their inputs have arisen^65^^,^ 97. As an illustration, consider proximal CA1 and distal subiculum neurons—they receive inputs from layer III MEC cells and send outputs to layer V/VI MEC cells. A similar pattern exists in the case of connections between distal CA1 and proximal subiculum neurons and the LEC.

In addition to reciprocal connections with the hippocampus, the LEC and the MEC also project to each other^26^.

### Dentate Gyrus

The dentate gyrus is a cortical region with a characteristic U or V shape, which lies proximal to the hippocampus, and possesses three cell layers^3^. It receives inputs from cells of the superficial layers of the EC via a pathway termed as the perforant pathway—so called because axons of the EC cells traverse through or perforate the subiculum as they go to the dentate gyrus^3^. The dentate gyrus, in turn, conveys this information to CA3 via axons termed as mossy fibres^7^. The dentate gyrus, like the hippocampus, possesses place cells. One of the unique features of the dentate gyrus is that new neurons are generated in this structure, even in adulthood (reviewed in Aimone et al.^1^).

### Hippocampus

The hippocampus is so called because the human hippocampus resembles the seahorse (genus: *Hippocampus*, itself named after the hippocamp, a mythical horse-fish hybrid). However, in rats, the main experimental organisms used in spatial memory studies, the hippocampus is a banana-shaped structure located in each cerebral hemisphere. The hippocampus consists of three cell layers as opposed to the neocortex, which contains six cell layers. The hippocampus is composed of three sub-regions, named CA1, CA2 and CA3^3^.

The connections to and from the hippocampus are complicated, not least because these differ amongst the CA1-3 sub-regions. Inputs to CA3 arise from layer II of the EC and the dentate gyrus. In contrast, CA1 receives inputs from layer III cells of the EC, and from CA3, but not directly from the dentate gyrus. In terms of outputs, CA3 neurons project on to CA1 neurons; in turn, CA1 sends outputs to the subiculum, and to the deeper layers (cell layers V and VI) of the EC^65^^,^ 97.

Having outlined the anatomy of the hippocampal formation (i.e., the hippocampus and its associated areas), we move to a description of some of the cell types that have been found to be part of this network. These cells are neural correlates of the computation involved in navigational strategies. It is important to note, though, that the list of cell types we have covered is not exhaustive, and only serves to indicate the diversity of cells that are involved in navigation.

## Neural Correlates of Navigation

### Place Cells

First identified in the rat hippocampus by O’Keefe and Dostrovsky^68^, place cells are neurons that exhibit a modulation in their firing rate, which is correlated with the spatial location of the animal. These cells, therefore, fire when an animal is at a particular position in an environment, and not, in general, at other positions in the environment; the region where the firing rate of the neuron is high is called the place field of the place cell. In typical experimental set-ups (up to 100 cm × 100 cm), place cells have been found to typically possess a single place field; however, in larger arenas ((150 cm × 140 cm) in Fenton et al.^30^; (180 cm × 140 cm) in Park et al.^70^) place cells possess several place fields.

Place cells have been primarily found in the hippocampus. However, there are also reports of cells with place-encoding properties from other brain regions, including the striatum^49^, the subiculum^79^ and the dentate gyrus^43^. Within the hippocampus, place cells have been found to be distributed in a non-topographic manner, i.e., place cells that are adjacent to each other do not have place fields that are adjacent to each other^67^^,^ 73. This is in contrast with cells from the visual and other sensory cortices, which are arranged in a topographic manner^42^^,^ 62 ^,^ 66. Furthermore, the size of place fields recorded in both CA1^44^ as well as CA3^45^ increases from the dorsal end of the hippocampus to the ventral end.

The position of place fields has been found to be governed by both idiothetic and allothetic cues. In the absence of sensory cues, only idiothetic cues are available to guide the firing of place cells. However, this does not assist in stable firing patterns^76^. This is consistent with the idea that errors that accumulate in the path integrator need to be corrected by taking a fix on landmarks to be able to consistently position oneself correctly in the environment. Altering the nature of allothetic cues in the environment of the animal can alter the firing patterns of the place cells—a phenomenon termed as remapping. Changes in sensory cues are not the only cause for remapping, however, as the motivational state^59^ and the behavioural context^53^ of the animal have also been found to be factors that influence this phenomenon. Two broad types of remapping are possible:

1. Rate remapping, in which the firing rate of place cells is altered (but not the positions of the place fields). This occurs when there is a change in the sensory cues present in a familiar environment; for example, a change in the colour of the walls^51^.
2. Global remapping, in which both the positions of the place fields and the firing rates of the place cells differ. It has been shown that changing the shape of an environment leads to an unpredictable change in the location of the place field(s)^51^^,^ 63.

Place cells have been subsequently discovered in other animals, including mice^55^, and bats 90; for place cells in three dimensions, see Yartsev et al.^99^.

The ensemble activity of place cells that are active in a given environment is considered to form a spatial map. The population vector formed by the firing rates of all instantaneously active place cells can be reliably used to deduce the instantaneous location of the animal^96^. Several models that build on this ensemble code have been proposed. One of them proposes that a pre-established synaptic network can encode several different environments uniquely if each environment is represented by the activity of a subset of place cells that are connected to each other^56^. CA3 pyramidal neurons project to other pyramidal neurons in CA3, forming a recurrent network^4^. This makes CA3 a suitable candidate for such a model. The model proposes that each time the rat enters a new environment, based on the sensory input, a subset of CA3 place cells is triggered and the firing pattern gets established immediately. If the probability of firing of a place cell is low and if it can fire in multiple environments, then such a network can encode a large number of environments^56^. Thus, this model posits that the system can encode multiple environments independently using a spatial network that gets established during brain development. An alternative model proposes that such a map can be formed using a simple synaptic learning rule to set the synaptic strengths of the recurrent connections in CA3. When an animal enters an environment, the external inputs that feed into CA3 facilitate firing of a number of CA3 neurons. The synapses between these simultaneously activated neurons get potentiated through synaptic plasticity. At each location, as the external inputs differ, a different subset of neurons is active. Distinct environments have distinct activity patterns depending upon the information brought in by inputs feeding into CA3^89^. Thus, according to this model, the spatial map forms as a consequence of experience of the environment and does not use a pre-established network with fixed synaptic strengths.

Place cells, therefore, act together in an ensemble to encode space. The subset of place cells, that forms this ensemble, depends on the inputs that the hippocampus receives. These inputs bring in idiothetic as well as allothetic information. In the following sections, we focus on these sources of idiothetic and allothetic spatial information to the hippocampus.

### Idiothetic Information

#### Grid Cells

After the discovery of place cells in the hippocampus, it became imperative to record from its inputs (like the EC) to understand how the hippocampus transforms its inputs into a spatial map. Early attempts to record from the EC (EC: Barnes et al.^5^; Frank et al.^32^; MEC: Quirk et al.^71^) demonstrated that it possessed weaker spatial specificity when compared to the hippocampus.

The first clear picture of the nature of spatial information encoded by the MEC emerged when a group led by Edvard Moser and May-Britt Moser targeted the dorsolateral MEC. Earlier studies had missed the dorsolateral MEC, and had, instead, targeted the intermediate MEC, which feeds into the intermediate hippocampus^61^. Pyramidal cells in the intermediate hippocampus have lower spatial resolution^44^^,^ 45 ^,^ 56. It is not surprising then, that the intermediate MEC (that projects to the intermediate hippocampus) should have lower spatial specificity. With the help of their collaborator Menno Witter, a neuroanatomist, the Mosers realised that the dorsolateral MEC feeds into the dorsal hippocampus, from where the most spatially selective place cells had previously been recorded^61^. Thus, knowledge of the anatomical connectivity of the two regions led them to discover position-modulated cells that showed discrete and regularly spaced place fields in the dorsal MEC^35^. Their subsequent study of the same region carried out in a larger experimental arena revealed that these cells form a repeating hexagonal or triangular grid-like pattern spanning the whole arena^40^. Such cells were consequently termed grid cells. The multiple firing fields of grid cells differ from those of place cells in that place cells have irregularly spaced firing fields in larger environments while grid fields are ordered.

In addition to the MEC, grid cells have been identified in both the presubiculum and the parasubiculum^8^. Besides this, cells exhibiting such properties have been found in a variety of animals, including mice^34^ and bats^100^.

The firing of grid cells, like that of place cells, is governed by both allothetic and idiothetic cues. Grid cells can maintain a stable firing pattern even in the darkness^40^, a fact that has led researchers to believe that these cells are involved in the processing of self-motion information. Also in contrast with place cells, the firing of grid cells is coordinated in that when the animal is introduced to a new environment, the firing patterns of the grid cells either rotate or shift coherently, maintaining a stable relationship with respect to each other^33^.

The periodic firing of a grid cell is thought to act like a graph paper and help measure distance between the start and the end point (in terms of the grid fields covered during the displacement). Grid cells have the same scale across various environments, and their firing persists even after the removal of landmarks. Both these properties point towards them being dependent on internally generated signals. Hence, they are hypothesized to play a role in path-integration^40^^,^ 61.

Models of grid cell formation use either attractor networks or oscillatory interference^37^; their details are beyond the scope of this review. It is relevant here, however, to think about how the brain can transform the information from the graph paper-like grid cell code to a place cell code that represents unique locations. One of the possible ways is through linear summation of synaptic inputs from grid cells of various scales and orientations, by hippocampal neurons. A place field can be formed wherever the vertices of multiple grid cells with a range of spatial scales sending strong synaptic inputs to a hippocampal neuron coincide^81^. Since this happens infrequently and in apparently irregular fashion, this can explain why place cells have one or a few, irregularly spaced, place fields under laboratory conditions. Savelli and Knierim^77^ showed how a simple synaptic plasticity rule modifying synaptic connections from grid to place cells can rapidly establish stable place fields in a novel environment.

If this model of generating place cells from grid cells indeed describes the entirety of mechanisms involved in place cell generation, disruption of grid cells will lead to disruption of place cells. Inactivation of medial septum leads to disruption of grid cells, but place cells continue firing at their preferred locations even in the absence of the periodic grid input, albeit with a reduced firing rate^47^. Thus, grid cell activity is not necessary for maintaining place cells. In contrast, inactivation of the hippocampus disrupts grid cells^9^. At the first glance, these two observations are incongruous with the grid cell to place cell models we discussed above. Does this mean that they are false and need to be discarded, or is there a more nuanced explanation? Inputs from the MEC to the hippocampus are complemented by inputs from the LEC. In the absence of inputs from grid cells, those from the LEC may be sufficient for maintaining place fields (see Sect. 3.3 for information conveyed by the LEC to the hippocampus). This could explain why grid cell disruption leads to a reduction in the firing rate, but not a loss of spatial selectivity, in the hippocampus. In this formulation, in conditions under which landmark derived information from the LEC is unreliable or unavailable, disruption of grid cell inputs would lead to more drastic disruption of place cells. The hippocampus sends strong feedback to the MEC. This can conceivably lead to a loop in which a grid cell plays a role in assigning a spatial location to a place cell, but in turn gets feedback from the hippocampus which helps stabilize/anchor the grid. Hence, while simple feedforward models might be easy to understand, the complex maze of neural interconnectivity might hold the key to complete understanding of information processing in the entorhinal–hippocampal network. We also need to take into account the lateral interactions between the LEC and the MEC (see Sects. 2.1 and 4) to understand this network in its entirety.

The path integration process, thought to give rise to grid cells, requires velocity (direction and speed) to be integrated in order to estimate displacement. We discuss the correlates of these two inputs below.

#### Head Direction (HD) Cells

HD cells are neurons whose activity is modulated by the direction (in the horizontal plane) in which the animal’s head points^72^^,^ 86. Such cells fire maximally when the animal’s head faces their preferred direction. Away from their preferred direction, the cells may fire at lower frequencies or may not fire at all. This typically leads to the generation of a Gaussian-shaped firing distribution curve that is centred at the peak firing direction. Within a population of HD cells, there is equal representation of all directions, with no preference for any specific direction^86^.

The firing of HD cells has been found to be independent of the position of the animal within the experimental arena and the orientation of the rest of the animal’s body^86^. It is dependent, however, on both allothetic and idiothetic cues. Sensory cues have been found to influence the preferred firing direction of HD cells. A change in the angular position of a visual landmark in the environment leads to a near identical rotation in the preferred firing direction^87^. Rotating an olfactory cue causes the preferred firing direction to rotate in the direction of cue rotation but to a lesser extent^38^. This is true, however, only if the cues are stable. In the absence of any allothetic information, idiothetic cues (through path integration) can control the firing of HD cells, though there are limits to the accuracy that can be obtained using this approach^101^.

The first HD cells were found in the rat dorsal presubiculum (also called the postsubiculum)^72^^,^ 86 ^,^ 87, but since then, they have been shown in several other brain regions. These areas include subcortical nuclei like the dorsal tegmental nucleus (which receives inputs from the vestibular system)^6^ and the lateral mamillary nuclei^83^ as well as the downstream recipients of signals from these two regions, including the anterodorsal thalamic nucleus^84^, the striatum^95^ and the MEC^75^.

Cells that have similar properties have been found in the presubiculum of monkeys^74^ as well. Cells responding to HD in 3-D space have been discovered in the presubiculum of bats^31^.

#### Speed Cells

The source of information on speed was a mystery till recently. The firing rate of grid cells in the MEC was found to be correlated with the speed of the animal^75^. Consequently, the Mosers led a study that identified cells (in both the MEC and the hippocampus) that showed a positive correlation between firing rate and running speed^48^. Though a significant population of these speed cells in the hippocampus turned out to be place cells with speed modulation, speed cells formed a distinct pool of their own in the MEC.

The speed signal was found to be independent of visual inputs from the environment, implying that at least part of it was derived from internally generated motion related cues. Speed cells also showed modulation by acceleration. When the rat accelerated, the firing rate of some speed cells also increased, and this rate was found to be correlated with the speed of the animal (albeit after a temporal lag). In other words, speed cells were identified as encoding future speed (i.e., prospective coding) rather than instantaneous speed, since the firing rate of the cells increased before there was an increase in the speed of the animal.

### Allothetic Information

So far, we have reviewed putative neural correlates of idiothetic information and path integration process. We will next look at the allothetic information flowing into the hippocampus, as it is thought to complement the idiothetic information in enabling hippocampal spatial representation by anchoring it to the real-world and acting as an error correction mechanism.

#### Object-responsive Cells

While the role of the MEC was being established, LEC was known to have object-related activity^103^, but its role in spatial navigation was untested. Recording LEC activity in spatial paradigms showed that the LEC has lesser spatial selectivity as compared to the MEC in standard spatial tasks in simple environments^41^ as well as cue-rich ones^102^. This is consistent with the hypothesis that the LEC conveyed nonspatial information to the hippocampus, while the MEC conveyed spatial information. Furthermore, as predicted by this hypothesis, when rats were trained to forage in an environment with objects placed at specific locations, LEC neurons showed an object-sensitive response. Some LEC neurons also fired in the presence of novel objects, while some fired when the location of one of the familiar objects was changed. Unexpectedly, some neurons showed place cell-like activity in the presence of objects^22^. The demonstration of conditional representation of spatial information in the LEC has prompted a change in the hypothesized role of the LEC, and the proposed LEC-MEC dichotomy. A modified hypothesis has now been put forth that the LEC encodes spatial as well as nonspatial information derived from external sensory stimuli, while the MEC encodes the product of path integration processes dependent on idiothetic information^21^^,^ 22.

#### Landmark Vector Cells

Collett and colleagues^15^ performed experiments that explored how gerbils could use landmarks for navigation. They trained groups of gerbils for various search tasks that involved using strategically placed objects to locate a goal (food reward) and discovered that the gerbils learn the relationship between a landmark and the goal, the geometric relationships between multiple landmarks and the relation of landmarks with the environment. While the geometric relationships between landmarks were used for matching landmarks to their stored representation, the goal locations appeared to be coded as a function of distance and direction from individual landmarks.

This ability of gerbils to localize a goal using landmarks can be explained by a vector encoding model proposed by McNaughton et al.^57^ They proposed that landmark vectors (LV) encode spatial locations as a function of distance and direction from a landmark. The magnitude (length) of the LV of an animal is the distance between the animal’s current location and the landmark, while the angle of the LV is measured with respect to an allocentric north (similar to that used by the HD system), which remains fixed for a given environment.

In this formulation, a place field of a place cell can be defined by a LV to the location of the place field, specifying that location using distance and direction from a landmark. A prerequisite for generating LVs is an environment in which landmarks and boundaries remain fixed. In such an environment, it is possible to have place cells with firing fields that are either anchored to an abstract reference frame not originating at any of the landmarks, or that are anchored to a landmark using a LV. It is not possible to distinguish between these two classes of cells, unless cells of the second category possess more than one place field, and each of these fields is anchored to a distinct landmark. The McNaughton et al.^57^ model predicts that a landmark vector cell “may become bound to one or more landmarks (typically one, occasionally two, rarely *more* than two)”. Deshmukh and Knierim^24^ used CA1 place cells with two or more fields to demonstrate the existence of neurons that encode two or more spatial locations as a function of distance and direction from two or more landmarks.

Landmark vector cells exemplify confluence of the two streams of information entering the hippocampus. They require information about the landmark (e.g. identity of the landmark) as well as distance and directional information from the idiothetic path integration system.

## Interplay Between Path Integration and Landmark-based Navigation

The two navigational strategies (path integration and landmark-based navigation) do not function in isolation—they are used to complement each other in correcting for defects and can also help in initial calibration of the navigation system. We highlight these interactions by first focusing on the use of landmark-based navigation to correct for errors in navigation arising from using only path integration. Given that the path integration system is prone to accumulating errors, it would be expected that animals that use path integration attempt to improve the accuracy of their navigation by using cues derived from landmarks. In other words, animals are expected to use landmarks to correct for inaccuracies in their navigation arising from the use of a purely path integration-based system. In this process, the path integrator would be reset to its correct value so that it can be used for the next leg of the journey.

Several studies conducted on a variety of animals support this hypothesis. For instance, it appears that insects associate local displacement vectors of specific directions and magnitudes (which together sum up to give the entire path taken by the animal) with landmarks (for details on bees, see Srinivasan et al.^82^; Collett et al.^14^; for ants, see Collett et al.^17^). Consequently, when such an insect passes a particular landmark, it undertakes a displacement corresponding to the specific vector^16^. The shorter the size of the displacement vector between two landmarks, the lesser is the extent of error accumulation in the path integration system during traversal of the path, and so the greater is the accuracy of navigation. By dividing the journey into smaller fractions, each of which is defined by a landmark, insects are able to navigate with increased accuracy.

In a different study, desert ants (*Cataglyphis fortis*) were captured at the end of an outward foraging trip and were either released at the entrance of the nest or forced back inside their nests (in both cases, without them having had to run back along the homebound tracks)^46^. In the first case, the ants ran along a vector path having the same magnitude and direction as the vector they would have used to navigate home from the feeding site, and consequently, these ants moved along a path that was opposite in direction to the path taken to get to the feeding centre. In contrast, ants that had been forced into their nests, on emerging from these nests, departed in the direction of the feeding site, indicating that they had reset their path integrator, possibly using cues located within the nest.

This trend appears to extend to mammals as well. Golden hamsters, which primarily use path integration in short excursions, have been found to use cues derived from brief exposures to stable, familiar visual landmarks to determine their azimuth, when their outward trip is carried out in darkness. By studying the view of landmarks in their vicinity, the hamsters update their position in a new reference frame, and thus, reset their path integrator^28^.

Rats appear to correct for heading errors (i.e., errors that accumulate in their HD signal) during a homing task by one of two strategies^92^. If the error is small, then the preferred firing direction of HD cells is reset to values that appear to be fixed with respect to the refuge in which the animal rested between trips. However, if the error is large, then the HD system is ‘remapped’, to acquire a new reference frame that remains stable for subsequent sessions.

We now proceed to understand the way in which path integration complements landmark-based navigation. Unfortunately, this has still not been explored in detail. Calibration of an animal’s landmark-based navigational system is important if the animal has never encountered a particular environment. In such an unfamiliar environment, the animal is solely dependent on navigational information derived from path integration and associates this information with landmarks in the environment, to become familiar with the area^16^. Studies on *Cataglyphis cursor* and *Cataglyphis fortis* have shown that that dead reckoning helps in learning landmarks only if the landmark is along the path that the animal takes to reach its goal. Landmarks located beyond the goal are not learnt by the animal, as they do not help the animal localize the goal itself. Such landmarks do not feature among the set of landmarks that it uses to navigate in that area^78^. However, no study conducted till now has examined the specific role of path integration in calibrating the landmark-based navigation system.

A small thought experiment can help test this interaction. Consider an experimentally naïve rat that is being allowed to navigate in a large arena that contains a single cue card attached to one of the walls of the arena. To use this cue card as a landmark, the rat needs to be able to determine how far it is from the cue card at every point in time, which is possible only if it has an estimate of the height/width of the card. From this value, as well as the visual angle subtended by the cue card, the rat can determine its position through simple trigonometry. However, the rat has not previously seen such a cue card (or the arena), and so, it has no expectation of the height/width of the cue card. In this case, the rat could run towards the cue card and integrate its velocity during this journey (through the path integration system) to arrive at a measure of its initial distance from the card. This estimate, along with the visual angle subtended by the object when the rat was at its initial position, could help it to get a measure of the height/width of the cue card, and this calibration could be carried out over multiple runs to increase the accuracy of the size estimate.

Besides this, recalibration of the landmark-based navigation system might be needed when the size of one or more of the landmarks in the environment is drastically altered. Our hypothesis that path integration could be used to calibrate landmark-based navigation can be tested in this scenario. Animals could be trained to perform spatial localization tasks that require use of one or a few landmarks, such as foraging for a food reward at a specific distance from the landmark(s). Once they are familiar with both the task and the environment, the size of the landmark(s) could be altered. In this situation, the animal does have an expectation about the size of the landmark(s), and so it may perform the task with its original estimate of the size of the landmark(s). Experiments on the European honey bee (*Apis mellifera*) in which a food source was kept near a landmark in the training session demonstrate this phenomenon. These studies have shown that bees go closer to the location where the food was kept during the training session, when the size of landmark is reduced. If the landmark is larger in size than that with which they were trained, they stop farther away from the location that they had been trained to land on^12^. Changing the size of landmark(s) as well as the environment may also affect neural correlates. Place fields in a cylinder with a cue card scale up with the cylinder and the cue card^63^. While this is consistent with our hypothesis, since both the cue card as well as the cylinder is enlarged, the relative contributions of the two are uncertain. An experiment conducted in a large arena with only the size of the landmark changing can enable us to study immediate rescaling of the spatial map in response to landmark size change, as well as reestablishment of the original scale of the map as the animal recalibrates its estimate of landmark size (possibly using path integration). If MEC lesioned rats are incapable of recalibrating the landmark-based navigation system in this task, it would provide evidence for the hypothesis that the path integrator is essential for calibrating the landmark system.

### Summary and Future Directions

In this review, we outlined various modes of navigation that are based on two primary navigational strategies observed in animals—path integration, and landmark-based navigation. Knowing that the hippocampal formation is one of the primary regions involved in navigation, we briefly covered its anatomy and then proceeded to look at some known neural correlates of navigation. Further, we tried to understand how these correlates relate to the strategies discussed earlier (for a more exhaustive review of spatially modulated cell types, refer to Grieves and Jeffrey^39^). Additionally, we discussed two ways in which these strategies interact with each other. Errors that accumulate in path integration have been found to be corrected by the infrequent use of landmarks (in taking a fix) to reset the path integrator. Besides this, we demonstrated that it is theoretically possible that the landmark-based navigation system could be calibrated using the path integration system. We also proposed experiments that could be used to verify this.

There are many questions that remain unanswered till date. How does the animal decide which strategy to use? How is the switch in strategies brought about, and what factors does this shift depend on?

Behaviour studies till date have been undertaken on various animals like mammals, insects and birds. The neural correlates we discussed have been recorded mostly from rodents and other mammals such as bats. The nervous system of any insect is quite different from that of a mammal; nevertheless, it uses the same strategy for navigation as do mammals. How do these species achieve similar computations?

Solving questions such as these will involve uniting the two traditionally distinct disciplines of neurophysiology and ethology. The result could enable us to obtain physiological data for a variety of animals and relate this with the feats of navigation they perform.
